# A discriminatory test for the wheat B and G genomes reveals misclassified accessions of *Triticum timopheevii* and *Triticum turgidum*

**DOI:** 10.1371/journal.pone.0215175

**Published:** 2019-04-10

**Authors:** Beata I. Czajkowska, Hugo R. Oliveira, Terence A. Brown

**Affiliations:** School of Earth and Environmental Sciences, Manchester Institute of Biotechnology, University of Manchester, Manchester, United Kingdom; Institute of Genetics and Developmental Biology Chinese Academy of Sciences, CHINA

## Abstract

The tetraploid wheat species *Triticum turgidum* and *Triticum timopheevii* are morphologically similar, and misidentification of material collected from the wild is possible. We compared published sequences for the *Ppd-A1*, *Ppd-B1* and *Ppd-G1* genes from multiple accessions of *T*. *turgidum* and *T*. *timopheevii* and devised a set of four polymerase chain reactions (PCRs), two specific for *Ppd-B1* and two for *Ppd-G1*. We used these PCRs with 51 accessions of *T*. *timopheevii* and 20 of *T*. *turgidum*. Sixty of these accessions gave PCR products consistent with their taxon identifications, but the other eleven accessions gave anomalous results: ten accessions that were classified as *T*. *turgidum* were identified as *T*. *timopheevii* by the PCRs, and one *T*. *timopheevii* accession was typed as *T*. *turgidum*. We believe that these anomalies are not due to errors in the PCR tests because the results agree with a more comprehensive analysis of genome-wide single nucleotide polymorphisms, which similarly suggest that these eleven accessions have been misclassified. Our results therefore show that the accepted morphological tests for discrimination between *T*. *turgidum* and *T*. *timopheevii* might not be entirely robust, but that species identification can be made cheaply and quickly by PCRs directed at the *Ppd-1* gene.

## Introduction

Wild and cultivated wheats comprise an allopolyploid complex of diploid (AA genomes), tetraploid (AABB and AAGG) and hexaploid forms (AABBDD and AAAAGG). The AABB species is called *Triticum turgidum* L. and includes wild and cultivated emmer (subsp. *dicoccoides* [Korn. ex Asch. & Graebn.] Thell. and subsp. *dicoccum* [Schrank ex Schübl.] Thell., respectively), both of which are hulled, meaning that the kernels are enclosed in toughened husks called glumes. Additionally, there is a series of cultivated emmer derivatives such as durum wheat (subsp. *durum* [Desf.] Husn.) and rivet wheat (subsp. *turgidum* (Desf.] Husn.), which are called naked or free-threshing wheats because they have thinner glumes that enclose the kernels less tightly. The AAGG tetraploid, *T*. *timopheevii* (Zhuk.) Zhuk., also has wild (subsp. *armeniacum* [Jakubz.] Slageren) and domesticated forms (subsp. *timopheevii*), both of which are hulled. *T*. *turgidum* and *T*. *timopheevii* can be crossed to produce F_1_ progeny (e.g. [1]), but these plants are sterile and the two species are thought to be non-interfertile due to failures in chromosome pairing [2].

The wild versions of *T*. *turgidum* and *T*. *timopheevii* have restricted geographical ranges, overlapping in southeast Turkey, northwest Syria and in the mountainous regions of eastern Iraq/western Iran, with *T*. *turgidum* additionally present in the upper Jordan valley and *T*. *timopheevii* in the Caucusus [3,4]. Although both species were domesticated by early farmers, only cultivated *T*. *turgidum* is considered to be a major crop, being grown extensively at Neolithic sites throughout the Fertile Crescent [3,5,6], and forming part of the package of crops whose cultivation spread into Europe, Asia and North Africa [3]. In contrast, *T*. *timopheevii* is looked on as a secondary crop, being found today only in western Georgia [3], although it has been suggested that the ‘new glume wheat’, which was grown by prehistoric farmers throughout western Asia and eastern Europe but is extinct today, might have been a form of *T*. *timopheevii* [7].

The hulled subspecies of *T*. *turgidum* and *T*. *timopheevii* have very similar morphologies and taxonomic identification is based mainly on the greater degree of hairiness of the culm internodes and leaf sheaths of *T*. *timopheevii* [8]. Misclassification is therefore possible, and DNA typing methods that can make unambiguous and correct identifications of the two species have been sought. However, identification of diagnostic DNA markers is complicated by the divergence time of the B and G genomes, which at 2.5–3.5 million years ago [9] is very recent in evolutionary terms, meaning that the two genomes share extensive DNA sequence identity. Additionally, in order to discriminate between *T*. *turgidum* and *T*. *timopheevii*, a marker must also give a null or diagnostic signal for the A genome, which diverged from the ancestor of the B and G genomes approximately 7 million years ago [9,10] and so also has extensive sequence similarity. Early studies indicated that the multicopy ribosomal DNA (rDNA) transcription units have features that enable the three genomes to be distinguished [11,12], and two polymerase chain reactions (PCRs) intended to be specific for the internal transcribed spacer of the G genome rDNA units were designed for identification of archaeological specimens [13]. However, one of these PCRs gave nonspecific amplification products with modern *T*. *turgidum* accessions and neither were successful with the ancient material. More recently, PCRs targeting chloroplast and mitochondrial DNA markers have been used [14,15], but these tests assume that the cytotype is an accurate proxy for the nuclear genome, which may not always be the case [14].

In order to identify nuclear markers for discrimination between *T*. *turgidum* and *T*. *timopheevii*, gene resequencing data (i.e. the sequences of orthologous genes from multiple accessions of the two species) are required so that species-specific sequence variations can be identified. The wheat gene for which the greatest amount of resequencing data is available is *Ppd-1*, coding for the major photoperiod response protein, with complete sequences in Genbank for 74 copies of *Ppd-B1*, 16 *Ppd-G1*, and 93 *Ppd-A1* (77 from *T*. *turgidum* and 16 from *T*. *timopheevii*) [16,17]. From this information we designed two PCRs that are specific for *Ppd-B1* and another two specific for *Ppd-G1*. Through use of these PCRs, we identify germplasm accessions of *T*. *turgidum* that have been misclassified as *T*. *timopheevii*, and vice versa.

## Materials and methods

Accessions of *T*. *turgidum* L. subsp. *dicoccoides* (Korn. ex Asch. & Graebn.) Thell., *T*. *turgidum* L. subsp. *dicoccum* (Schrank ex Schübl.) Thell., *T*. *timopheevii* (Zhuk.) Zhuk. subsp. *armeniacum* (Jakubz.) Slageren and *T*. *timopheevii* (Zhuk.) Zhuk. subsp. *timopheevii* (S1 Table) were obtained from: the Centre for Genetic Resources (CGN), Wageningen, Netherlands; the International Center for Agricultural Resources in the Dry Areas (ICARDA), Beirut, Lebanon; the Leibniz Institute of Plant Genetics and Crop Plant Research (IPK), Gatersleben, Germany; and the National Small Grains Collection (NSGC), Aberdeen, Idaho, USA. Seeds were germinated at room temperature (c.22°C) in Petri dishes in hydroponic conditions until coleoptiles emerged. Seeds were then transferred to moist filter paper and seedlings grown until 21 days old. Fresh leaf material was collected and DNA extracted using the ISOLATE II Plant DNA kit (Bioline).

DNA sequences were downloaded from Genbank for *Ppd-B1* from 24 accessions of *T*. *turgidum* subsp. *dicoccoides* and 50 *T*. *turgidum* subsp. *dicoccum*, *Ppd-G1* from 11 *T*. *timopheevii* subsp. *armeniacum* and 5 *T*. *timopheevii* subsp. *timopheevii*, and *Ppd-A1* from 32 *T*. *turgidum* subsp. *dicoccoides*, 45 *T*. *turgidum* subsp. *dicoccum*, 11 *T*. *timopheevii* subsp. *armeniacum* and 5 *T*. *timopheevii* subsp. *timopheevii* (S2 Table). Sequences were aligned using the ClustalW, Muscle and Mafft programs in Geneious version R10 (https://www.geneious.com, [18]) and single nucleotide polymorphisms (SNPs) that are specific to the different genomes identified. Primer pairs were identified for four PCRs (Table 1), two specific for *Ppd-B1* and two for *Ppd-G1*. PCRs were carried out in a LightCycler480 (Roche) in 20 μl reaction volumes comprising 100 ng DNA extract, 1x SensiFAST SYBR No-ROX PCR master mix (Bioline), 100 nM forward primer, 100 nM reverse primer and PCR grade water. Cycling parameters were: 95°C for 5 min; followed by 35 cycles of 20 s at 95°C, 20 s at the annealing temperature, 20 s at 72°C; followed by a final extension at 72°C for 10 min. Product formation was assayed using the SYBR Green I/HRM Dye detection format (465 nm excitation, 510 nm emission) by melt curve analysis. Melting data were obtained by heating the products to 95°C for 5 s, cooling to 55°C for 30 s and then heating to 99°C with five data acquisitions/°C. Melting peaks were obtained by plotting–(δF/δT) against temperature. PCR products were additionally visualized by electrophoresis in 3% agarose gels to confirm they were the correct length.

**Table 1:** Details of PCRs.

PCR	Primers	Annealing temperature (°C)	Product size (bp)	Specific for
**1**	Forward: 5´–TGAAGCACAGAGCAAACACC–3´	67	84	*Ppd-B1*
	Reverse: 5´–TTGATCACGTTGGACTGAGC–3´			
**2**	Forward: 5´–TCTGAAAGCCGATTTCGTTT–3´	66	100	*Ppd-B1*
	Reverse: 5´–GCACCTGCAAAAGGAATGAT–3´			
**3**	Forward: 5´–TGAACACAGACGGTCAGTCC–3´	64	61	*Ppd-G1*
	Reverse: 5´–CGTCCATTATCGGTTGGTTT–3´			
**4**	Forward: 5´–GGGAAGGAGCTGGAGATAGG–3´	67	69	*Ppd-G1*
	Reverse: 5´–ACTCTCATTCGGGGAGGACT–3´			

Prior to sequencing, PCR products were cloned (Invitrogen TOPO TA Cloning Kit for Subcloning, with One Shot TOP10 chemically competent *E*. *coli* cells) and reamplified, using the conditions described above except for the final extension at 72°C, with forward and reverse M13 primers (annealing temperature 55°C) and recombinant colonies added directly to the PCR mixture. PCR products were purified with the NucleoSpin Gel and PCR Clean-up kit (Macherey-Nagel) and sequenced using the BigDye Terminator v3.1 kit chemistry (Applied Biosystems). Standard sequencing reactions of 20 μl comprised 20 ng PCR product, 1x BigDye sequencing buffer, 0.125x BigDye reaction mix, 4 pmoles M13 primer and UltraPure DNase/RNase-free distilled water. Cycling parameters were: 2 min at 96°C; 35 cycles of 40 s at 96°C, 15 s at 50°C, 4 min at 60°C; with products held at 4°C before purification (Beckman Coulter Agencourt CleanSEQ kit) and reading of paired-end sequences by capillary electrophoresis in a 3730 DNA Analyser (Applied Biosystems).

Genotyping-by-sequencing (GBS) was carried out (Genomic Diversity Facility, Cornell University) with a panel of 138 tetraploid wheats comprising 76 *T*. *turgidum* subsp. *dicoccoides*, 43 *T*. *turgidum* subsp. *dicoccum*, 11 *T*. *timopheevii* subsp. *armeniacum* and 8 *T*. *timopheevii* subsp. *timopheevii*, using a standard method [19]. Unique sequence tags were aligned to release 31 of the genome of *Triticum aestivum* L. [20] using BWA v.0.7.8-r455 [21] and SNPs identified with the TASSEL-GBS pipeline [22]. Principal components analysis (PCA) was performed with TASSEL [23].

## Results

**Fig 1:**
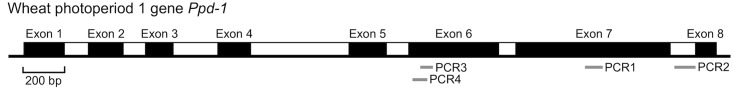
Schematic of the wheat *Ppd-1* gene. Exons are shown as closed boxes and introns as open boxes. The positions of the four PCRs are indicated.

The consensus sequence resulting from multiple alignment of the 173 *Ppd-1* Genbank entries had a total length of 7302 bp with the first nucleotide of the initiation codon at position 3604 and the last nucleotide of the termination codon at position 6819. The alignment was used to design two PCRs specific for *Ppd-B1*, one of these located within exon 7 of the gene and the second mainly in intron 7 but with its 3´–terminus extending a short distance into exon 8, and a further two PCRs specific for *Ppd-G1*, both of these targeting sequences within exon 6 (Fig 1). The PCRs were designed so that each primer pair had a 100% match with their annealing sites on the target genome, but at least two mismatches with the equivalent sites on the non-target genomes (Table 2). Each primer pair gave a single product of the expected size when used with DNA from its target species, and no product with the non-target species (S1 and S2 Figs), confirming the specificities of the PCRs.

**Table 2:** Differences between the primer sequences and the equivalent sequences on the non-target genomes.

**PCR1**	**Forward primer**	**Reverse primer**
**B genome (target sequence)**	TGAAGCACAGAGCAAACACC	GCTCAGTCCAACGTGATCAA
**G genome**	TGAAGCACAGAGCAAACA**T**C	GCTCAGTCCA**GTT**TG**G**TCAA
**A genome**	TGAAGCACAGAGCAAACACC	GCTCAGTCCA**GTT**TG**G**TCAA
**PCR2**	**Forward primer**	**Reverse primer**
**B genome (target sequence)**	TCTGAAAGCCGATTTCGTTT	ATCATTCCTTTTGCAGGTGC
**G genome**	**–**C**CT**AAAGCCG**TC**TT**G**GT**C**T	**G**TCATT**GA**TTTT**T**CAGGTGC
**A genome**	**–**C**C**GAAAGCCGATT**C**CGT**C**T	**G**T**A**A**C**TC**A**TTTTGCAGGTGC
**PCR3**	**Forward primer**	**Reverse primer**
**G genome (target sequence)**	TGAACACAGACGGTCAGTCC	AAACCAACCGATAATGGACG
**B genome**	TGAACACAGA**T**G**A**TCA**A**TCC	AAACCAAC**A**GATAATGGACG
**A genome**	TGAACACAGA**T**G**A**TCAGTCC^a^	AAACCAAC**T**GAT**–––**GGACG^a^
**PCR4**	**Forward primer**	**Reverse primer**
**G genome (target sequence)**	GGGAAGGAGCTGGAGATAGG	AGTCCTCCCCGAATGAGAGT
**B genome**	GGGAAGGAGCTGGAGATAGG	A**A**TCCTCCCCGAA**C**GAGAGT
**A genome**	GGGAAGGAG**T**TGGAGATAGG^a^	AGTCCTCCCCGAA**C**GAGAGT^a^

Differences between the sequences of the primers and the non-target genomes are shown in bold.

^a^ In some accessions of *T*. *turgidum* subsp. *dicoccum* the target sequence is absent due to a larger deletion in the *Ppd-A1* gene.

**Fig 2:**
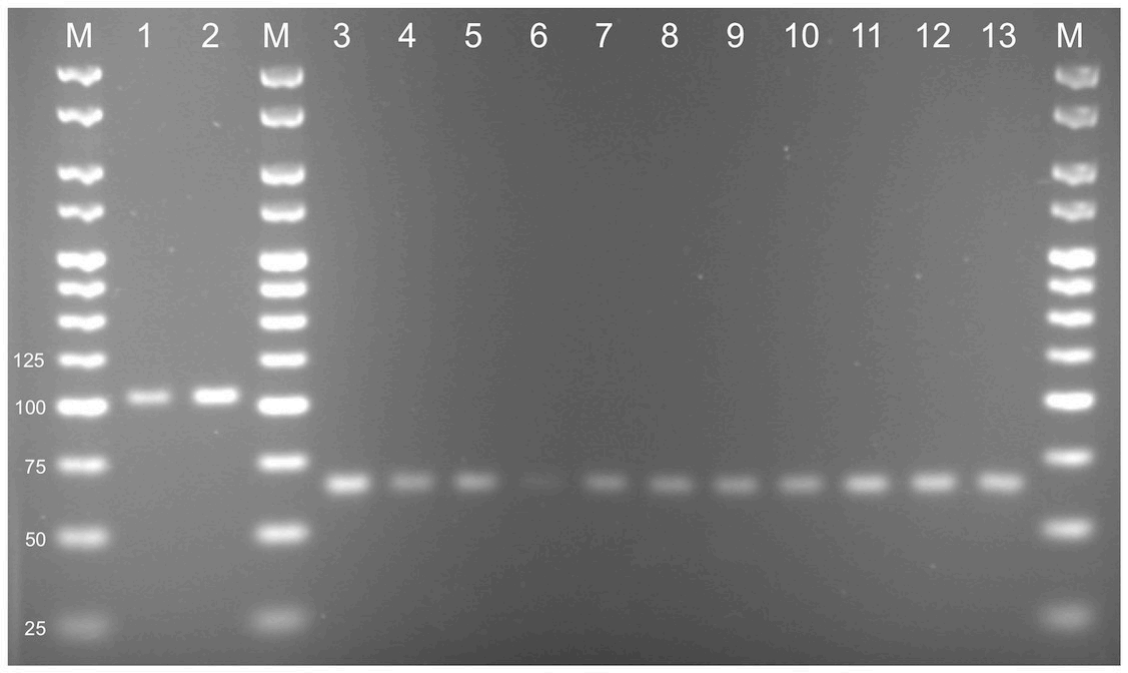
PCR products obtained from eleven anomalous accessions. Lanes 1 and 2: results of PCR2, specific for *Ppd-B1*, with PI 286061 (lane 1, authentic *T*. *turgidum* subsp. *dicoccum*) and PI 427998 (lane 2, classified as *T*. *timopheevii* subsp. *armeniacum*). Lanes 3–13: results with PCR3, specific for *Ppd-G1*, with PI 341802 (lane 3, authentic *T*. *timopheevii* subsp. *timopheevii*), PI 560697 (lane 4), PI 560873 (lane 5), PI 560877 (lane 6), PI 656869 (lane 7), PI 656872 (lane 8), PI 656873 (lane 9), CGN 16098 (lane 10), CGN 16102 (lane 11), CGN 13161 (lane 12) and CGN 24296 (lane 13) (all classified as *T*. *turgidum* subsp. *dicoccoides*). M, size markers (bp).

**Fig 3:**
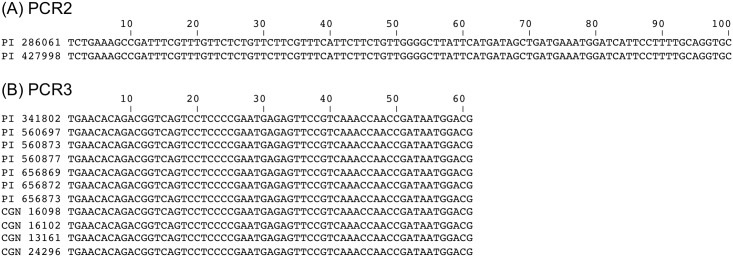
Sequences of PCR products obtained from eleven anomalous accessions. (A) PCR2, specific for *Ppd-B1*, with PI 286061 (authentic *T*. *turgidum* subsp. *dicoccum*) and PI 427998 (classified as *T*. *timopheevii* subsp. *armeniacum*). (B) PCR3, specific for *Ppd-G1*, with PI 341802 (authentic *T*. *timopheevii* subsp. *timopheevii*), PI 560697, PI 560873, PI 560877, PI 656869, PI 656872, PI 656873, CGN 16098, CGN 16102, CGN 13161 and CGN 24296 (all classified as *T*. *turgidum* subsp. *dicoccoides*).

The PCRs were used with 51 accessions of *T*. *timopheevii* and 20 of *T*. *turgidum* (S3 Table). Sixty accessions gave PCR products consistent with their taxon identifications. The other eleven accessions gave anomalous results (Table 3). These accessions comprised ten that were classified as *T*. *turgidum* subsp. *dicoccoides* but which gave positive results with the *Ppd-G1* but not the *Ppd-B1* PCRs, and which were therefore typed as *T*. *timopheevii*, and one *T*. *timopheevii* subsp. *armeniacum* accession which gave positive results for *Ppd-B1* but not *Ppd-G1*, and so was identified as *T*. *turgidum* (Fig 2). For each of these eleven anomalous accessions, the PCR products that were obtained were sequenced and their authenticity as *Ppd-B1* or *Ppd-G1* products confirmed from the presence of specific variations within the internal part of the amplicon (Fig 3).

**Table 3:** Accessions giving anomalous results after *Ppd-1* typing.

Accession number	Original classification	Species according to *Ppd-1* typing	Collection site		
			Country	Latitude	Longitude
**PI 560697**	*T*. *turgidum* subsp. *dicoccoides*	*T*. *timopheevii*	Turkey	37.58333	42.38333
**PI 560873**	*T*. *turgidum* subsp. *dicoccoides*	*T*. *timopheevii*	Turkey	37.47	42.03
**PI 560877**	*T*. *turgidum* subsp. *dicoccoides*	*T*. *timopheevii*	Turkey	38.13	41.26
**PI 656869**	*T*. *turgidum* subsp. *dicoccoides*	*T*. *timopheevii*	Turkey	37.2214	37.3303
**PI 656872**	*T*. *turgidum* subsp. *dicoccoides*	*T*. *timopheevii*	Turkey	37.2026	37.0925
**PI 656873**	*T*. *turgidum* subsp. *dicoccoides*	*T*. *timopheevii*	Turkey	37.1939	37.0944
**CGN 16098**	*T*. *turgidum* subsp. *dicoccoides*	*T*. *timopheevii*	Iran	37.28083	49.58306
**CGN 16102**	*T*. *turgidum* subsp. *dicoccoides*	*T*. *timopheevii*	Iraq	33.138	44.43333
**CGN 13161**	*T*. *turgidum* subsp. *dicoccoides*	*T*. *timopheevii*	Iraq	33.639	44.43333
**CGN 24296**	*T*. *turgidum* subsp. *dicoccoides*	*T*. *timopheevii*	Iraq	33.334	44.43333
**PI 427998**	*T*. *timopheevii* subsp. *armeniacum*	*T*. *turgidum*	Lebanon	33.51667	35.86667

**Fig 4:**
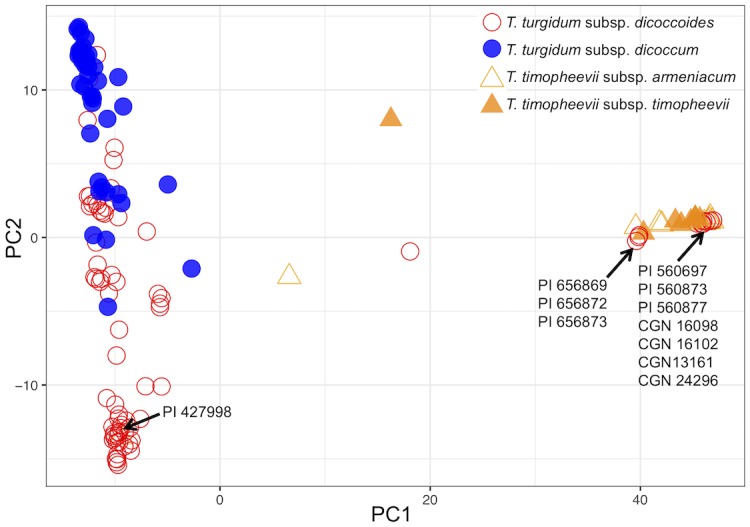
PCA of 138 tetraploid wheat accessions based on 1,172,469 SNPs obtained by GBS. The positions of the eleven anomalous accessions are indicated. PC1 accounts for 5.59% of the variance and PC2 accounts for 2.26%.

GBS was carried out with 138 tetraploid wheats including each of the eleven accessions that gave anomalous results by *Ppd-1* typing. The resulting dataset of 1,172,469 SNPs was examined by PCA. The first principal component (PC1) separated the *T*. *turgidum* and *T*. *timopheevii* accessions into distinct clusters (Fig 4). Each of the ten accessions classified as *T*. *turgidum* subsp. *dicoccoides* but identified as *T*. *timopheevii* by *Ppd-1* typing were positioned within the *T*. *timopheevii* cluster, and the single accession classified as *T*. *timopheevii* subsp. *armeniacum* but identified as *T*. *turgidum* by *Ppd-1* typing was located within the *T*. *turgidum* cluster.

## Discussion

We designed two PCRs specific for the *Ppd-B1* gene and two for *Ppd-G1* and tested these with 71 *T*. *timopheevii* and *T*. *turgidum* accessions. For 60 accessions, the results of the PCRs were consistent with the species identification, giving positive results for *Ppd-B1* and negative for *Ppd-G1*, or vice versa, indicating that the PCRs were specific for their target sequences and that neither of the PCRs gave products with the *Ppd-A1* gene on the A genome.

There were, however, eleven anomalous accessions, ten which gave positive results for *Ppd-G1* despite being classified as *T*. *turgidum*, and one classified as *T*. *timopheevii* that was typed positive for *Ppd-B1*. Previous contradictions between the outcomes of PCR typing and the morphological identification of a wheat as *T*. *timopheevii* or *T*. *turgidum* have been dismissed as errors in the DNA method [17]. However, we believe that with the anomalies we report our DNA typing results are correct and the accessions have previously been misclassified. This is because each of these eleven accessions were included in a larger group of 138 *T*. *timopheevii* and *T*. *turgidum* wheats for which we obtained GBS data. PCA of the resulting SNPs separated the 138 accessions into two clusters, one cluster comprising *T*. *timopheevii* wheats plus the ten accessions that were classified as *T*. *turgidum* but which gave a positive result for *Ppd-G1*, and the second cluster made up of *T*. *turgidum* plus the one accession that was classified as *T*. *timopheevii* but which gave a *Ppd-B1* result. As the SNPs used in the PCA mapped to all 14 tetraploid wheat chromosomes, with >59,000 markers per chromosome, we can be confident that the clustering reflects genome-wide differences between the groups of accessions, and therefore is giving an accurate identification of whether each wheat has an AABB or AAGG genome set. The agreement between the PCAs and the *Ppd-1* typing therefore confirms that these eleven accessions have been misclassified, and that *Ppd-1* typing (which is much less time-consuming and costly than GBS analysis) is an accurate means of distinguishing between *T*. *timopheevii* and *T*. *turgidum*.

The entries for the eleven misclassified accessions in the Germplasm Resources Information Network (GRIN) and the European Wheat Database (EWDB) give no indications that the original material that was collected might have been misidentified. However, the ten accessions misclassified as *T*. *turgidum* were collected from Turkey, Iran and Iraq, which are within the distribution range for wild *T*. *timopheevii*, and the one misidentified as *T*. *timopheevii* was collected in the Lebanon, which is outside of the area normally associated with *T*. *timopheevii* [3]. Three of the accessions misidentified as *T*. *turgidum* (PI 560697, PI 560873 and PI 560877) were previously reclassified by us as *T*. *timopheevii* based on the pattern of retrotransposon insertions in the 5S rDNA arrays [24], and two (PI 560697 and PI 560877) were similarly classified as *T*. *timopheevii* in a study of the grain *Hardness* locus [25]. In contrast, PI 560697 was included in a panel of 113 wild *T*. *turgidum* accessions used in a survey of allelic diversity at the ear-shattering loci, *TtBtr1-A* and *TtBtr1-B* [26], although PI 560697 gave an unusual result, being one of only two accessions that possessed the domesticate allele at *TtBtr1-A*. None of the other seven accessions that we reclassify as *T*. *timopheevii* (PI 656869, PI 656872, PI 656873, CGN 16098, CGN 16102, CGN 13161, CGN 24296) appear to have been extensively studied in the past. The single accession that we reclassify from *T*. *timopheevii* to *T*. *turgidum* (PI 427998) was listed as *Triticum boeoticum*, a wild diploid wheat, now called *Triticum monococcum* L. subsp. *aegilopoides* (Link) Thell., in a study of molecular diversity at 18 genetic loci [27], but was subsequently looked on as *T*. *turgidum* in the retrotransposon and *Hardness* projects mentioned above [24,25].

## Conclusion

We show that the *Ppd-1* gene of wheat displays species-specific variations that enable the B and G genomes to be distinguished via simple PCR tests, the outcomes of these tests agreeing with identifications made by more comprehensive, but more time-consuming and expensive, analysis of genome-wide SNPs. The use of *Ppd-1* typing reveals a significant number of misclassified accessions, in particular wheats initially identified as *T*. *turgidum* but which we show to be *T*. *timopheevii*, suggesting that the accepted morphological tests for discrimination between the two species might not be entirely robust. The short lengths of the amplicons (61–100 bp) means that the tests we report would be particularly suitable for typing ancient DNA, which is typically obtained as fragments <100 bp [28]. Among other archaeological applications, these tests might therefore make it possible to establish if the new glume wheat [7] is a type of *T*. *turgidum* or *T*. *timopheevii*.

## Supporting information

S1 Fig Agarose gel showing products of B- and G-specific PCRs. Within each set of four lanes the PCR has been carried out with (left to right) *T*. *timopheevii* subsp. *timopheevii* PI 341802, *T*. *timopheevii* subsp. *armeniacum* Cltr 17678, *T*. *turgidum* subsp. *dicoccum* PI 286061, *T*. *turgidum* subsp. *dicoccoides* PI 428143. Lanes 1, 10 and 19 are DNA size markers.
(TIFF)

S2 Fig Melting peaks of products of B- and G-specific PCRs. (A) PCR1 with *T*. *turgidum* subsp. *dicoccum* PI 286061 and *T*. *turgidum* subsp. *dicoccoides* PI 428143; (B) PCR2 with *T*. *turgidum* subsp. *dicoccum* PI 286061 and *T*. *turgidum* subsp. *dicoccoides* PI 428143; (C) PCR3 with *T*. *timopheevii* subsp. *timopheevii* PI 341802 and *T*. *timopheevii* subsp. *armeniacum* Cltr 17678; (D) PCR4 with *T*. *timopheevii* subsp. *timopheevii* PI 341802 and *T*. *timopheevii* subsp. *armeniacum* Cltr 17678. The blue lines are no-template controls. Melting peak analysis enables PCR specificity to be confirmed because products with different sequences melt at different temperatures. A single peak therefore indicates that a single PCR product has been formed.
(TIFF)

S1 Table Wheat accessions used in this study. (XLSX)

S2 Table Genbank entries for *Ppd-1* used in design of PCRs. (XLSX)

S3 Table Results of PCRs. (XLSX)
