# Supplementary figures and images for: A discriminatory test for the wheat B and G genomes reveals misclassified accessions of Triticum timopheevii and Triticum turgidum
Source: PLoS One. 2019 Apr 10;14(4):e0215175. doi: 10.1371/journal.pone.0215175 (PMC6457550; doi:10.1371/journal.pone.0215175)

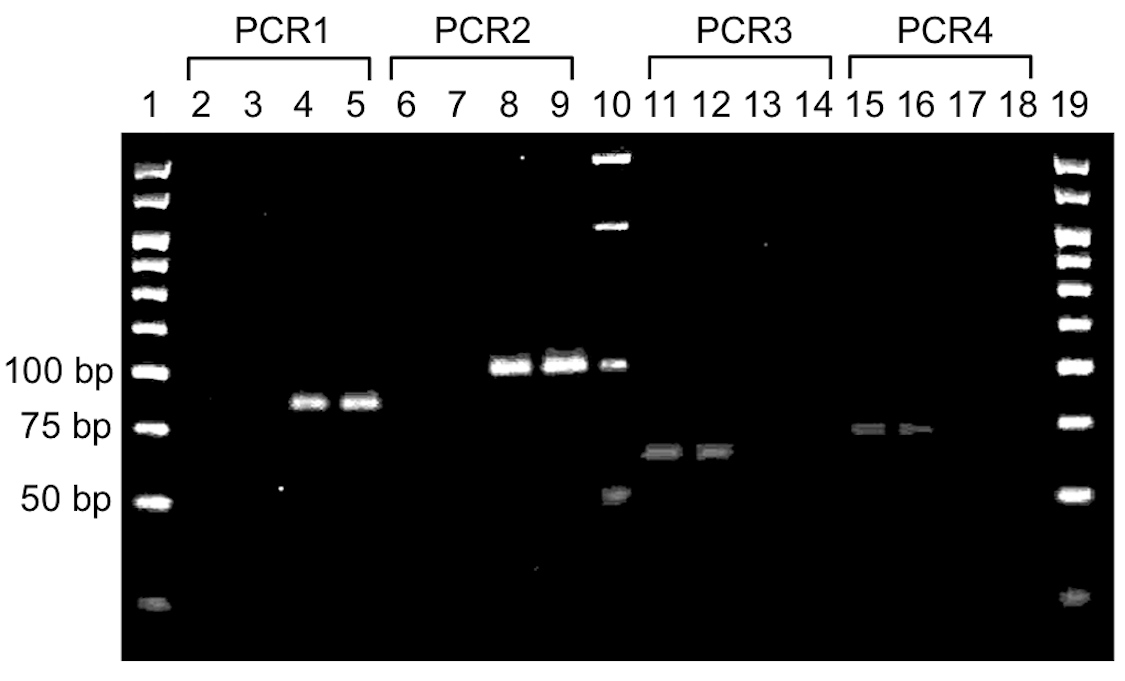

Supplement: S1 Fig — Within each set of four lanes the PCR has been carried out with (left to right) T. timopheevii subsp. timopheevii PI 341802, T. timopheevii subsp. armeniacum Cltr 17678, T. turgidum subsp. dicoccum PI 286061, T. turgidum subsp. dicoccoides PI 428143. Lanes 1, 10 and 19 are DNA size markers. (TIFF) [file pone.0215175.s001.tiff]

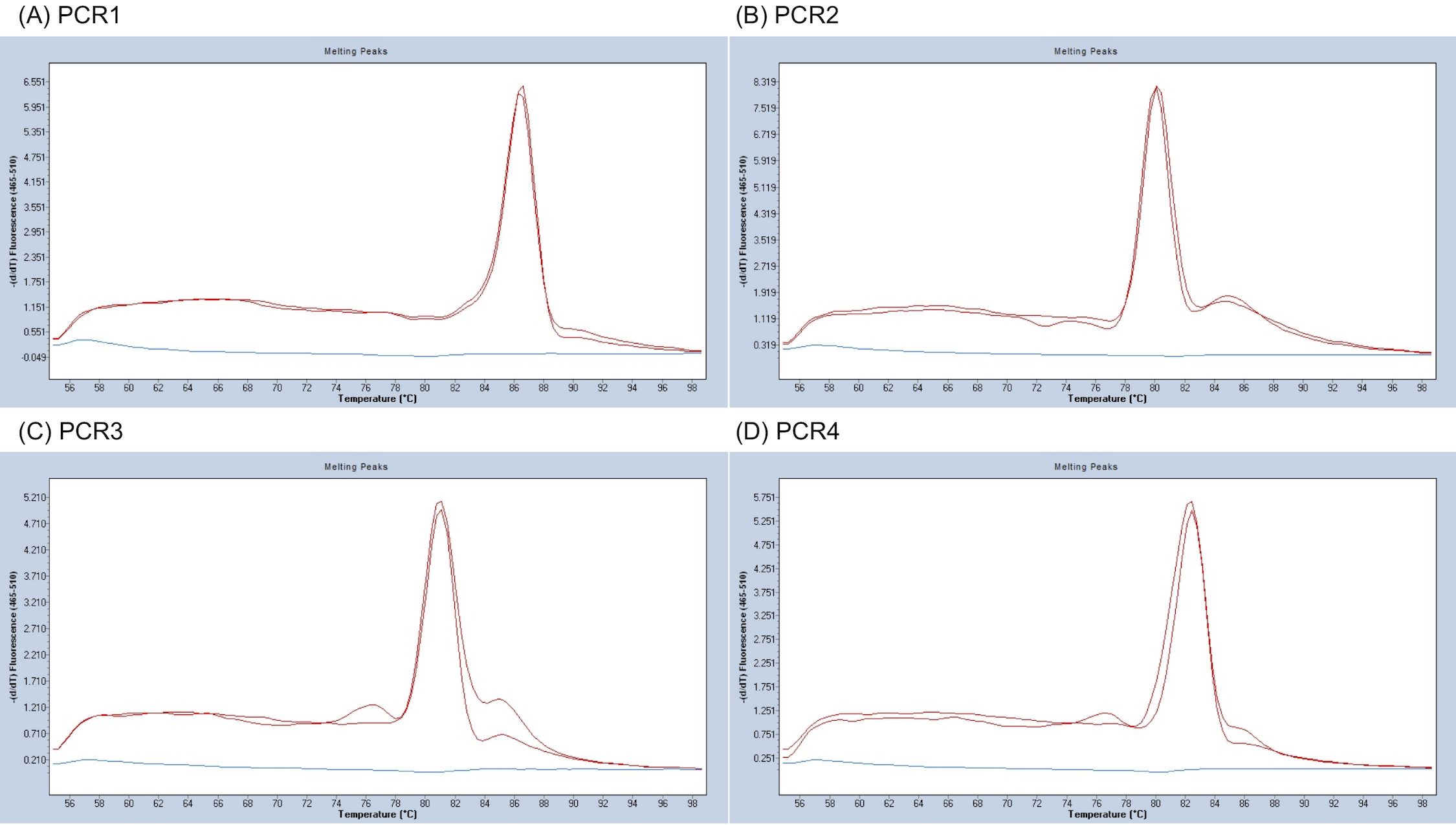

Supplement: S2 Fig — (A) PCR1 with T. turgidum subsp. dicoccum PI 286061 and T. turgidum subsp. dicoccoides PI 428143; (B) PCR2 with T. turgidum subsp. dicoccum PI 286061 and T. turgidum subsp. dicoccoides PI 428143; (C) PCR3 with T. timopheevii subsp. timopheevii PI 341802 and T. timopheevii subsp. armeniacum Cltr 17678; (D) PCR4 with T. timopheevii subsp. timopheevii PI 341802 and T. timopheevii subsp. armeniacum Cltr 17678. The blue lines are no-template controls. Melting peak analysis enables PCR specificity to be confirmed because products with different sequences melt at different temperatures. A single peak therefore indicates that a single PCR product has been formed. (TIFF) [file pone.0215175.s002.tiff]
